# Thrombin acts as inducer of proinflammatory macrophage migration inhibitory factor in astrocytes following rat spinal cord injury

**DOI:** 10.1186/s12974-022-02488-w

**Published:** 2022-05-27

**Authors:** Ting Yang, Haiyan Jiang, Xinye Luo, Yuxuan Hou, Aicheng Li, Bingqiang He, Xingyuan Zhang, Huifei Hao, Honghua Song, Rixin Cai, Xudong Wang, Yingjie Wang, Chun Yao, Lei Qi, Yongjun Wang

**Affiliations:** 1 Key Laboratory of Neuroregeneration of Jiangsu and Ministry of Education, Co-Innovation Center of Neuroregeneration, Nantong University, 19 Qixiu Road, Nantong, 226001 People’s Republic of China; 2 Health Management Center, Affiliated Hospital of Nantong University, Nantong, 226001 People’s Republic of China; 3 Department of Emergency Medicine, Affiliated Hospital of Nantong University, Nantong, 226001 People’s Republic of China; 4 Department of Laboratory Medicine, Affiliated Hospital of Nantong University, School of Public Health, Nantong University, Nantong, 226001 Jiangsu People’s Republic of China

**Keywords:** Thrombin, MIF, CNS, Astrocyte, Inflammation, NFκB, PAR receptor, Spinal cord, MAPKs, DAMPs

## Abstract

**Background:**

The danger-associated molecular patterns (DAMPs) are critical contributors to the progressive neuropathology and thereafter affect the functional outcomes following spinal cord injury (SCI). Up to now, the regulatory mechanisms on their inducible production from the living cells remain elusive, aside from their passive release from the necrotic cells. Thrombin is immediately activated by the damaged or stressed central nervous system (CNS), which potently mediates inflammatory astrocytic responses through proteolytic cleavage of protease-activated receptors (PARs). Therefore, SCI-activated thrombin is conceived to induce the production of DAMPs from astrocytes at lesion site.

**Methods:**

Rat SCI model was established by the cord contusion at T8–T10. The expression of thrombin and macrophage migration inhibitory factor (MIF) was determined by ELISA and Western blot. The PAR1, PAR3, and PAR4 receptors of thrombin were examined by PCR and immunohistochemistry. Primary astrocytes were isolated and purified from the spinal cord, followed by stimulation with different concentrations of thrombin either for transcriptome sequencing or for analysis of thrombin-mediated expression of MIF and related signal pathways in the presence or absence of various inhibitors. The post-injury locomotor functions were assessed using the Basso, Beattie, and Bresnahan (BBB) locomotor scale.

**Results:**

MIF protein levels were significantly elevated in parallel with those of thrombin induced by SCI. Immunostaining demonstrated that PAR1 receptor, together with MIF, was abundantly expressed in astrocytes. By transcriptome sequencing and bioinformatical analysis of thrombin-stimulated primary astrocytes, MIF was identified to be dynamically regulated by the serine protease. Investigation of the underlying mechanism using various inhibitors revealed that thrombin-activated PAR1 was responsible for the MIF production of astrocytes through modulation of JNK/NFκB pathway. Administration of PAR1 inhibitor at lesion sites following SCI significantly reduced the protein levels of MIF and ameliorated functional deficits of rat locomotion.

**Conclusion:**

SCI-activated thrombin is a robust inducer of MIF production from astrocytes. Exploring the roles of thrombin in promoting the production of DAMPs from astrocytes at lesion site will provide an alternative strategy for the clinical therapy of CNS inflammation.

## Introduction

Spinal cord injury (SCI) will lead to a series of cellular and molecular changes at lesion site associating with the progressive neuropathology [1]. Early after SCI, the resident and infiltrating immune cells are rapidly activated and thereby release a plethora of inflammatory mediators to exacerbate the damaged milieu [2]. As a consequence, cell death, axonal degeneration, and demyelination will proceed in response to excessive inflammation, and persist for several days or longer before a glial/fibrotic scar forms at later time points [3, 4]. The magnitude of inflammation is well correlated with the extent of tissue damage and functional loss under regulation of complex signal cascades [5, 6]. It has been known that SCI-induced inflammatory response is awakened by danger-associated molecular patterns (DAMPs), which are passively released from necrotic cells or actively secreted by immune cells [2, 7]. DAMPs interact with the pattern recognition receptors (PRRs) of immune and/or non-immune cells in the central nervous system (CNS) to potentiate inflammatory signals by producing various proinflammatory mediators [2, 8–12]. Interference of these DAMPs by genetic manipulation or specific inhibitors significantly mitigates inflammatory response and promotes functional recovery following damage of CNS [13–15]. As such, the minimization of DAMPs production at the lesion epicenter is crucial for restricting the expansion of inflammation and alleviating the secondary neurodegenerative changes. Many cell types in the CNS can inducibly produce DAMPs in response to various insults, though at different yields [16–18]. Identification of the core mediator(s) that drives the DAMPs production is of great significance for the resolution of CNS inflammation.

Astrocytes are primary glial cell type in the CNS that contribute to maintenance of blood–brain (spinal cord) barrier (BBB or BSCB), provision of trophic and metabolic support for the neurons [1, 19, 20]. They also respond to various CNS insults with a response known as reactive astrogliosis [21–23]. The reactive astrocytes play active roles in neuroinflammation via dynamic secretion of cytokines and chemokines in addition to formation of glial scar [24]. Mechanism insights reveal that astrocytes express similar profile of PRRs to those of microglia, endowing them to activate innate immunity once sensing DAMPs [25]. In fact, DAMPs-induced activation of STAT1/3 and NFκB in astrocytes results in exacerbation of clinical signs and motor deficits in distinct disease models, due to aberrant production of proinflammatory mediators [26–28]. Therefore, reactive astrocytes are recognized as immune-competent cells aside from other pathological functions [24]. It is interesting to conceive whether astrocytes are associated with the active production of DAMPs in the damaged or diseased CNS. Unveiling the underlying mechanism will provide better understanding for the astrocytes-related neuroinflammation.

Macrophage migration inhibitory factor (MIF) is a pivotal regulator of innate immunity that plays a central role in inflammatory responses by promoting the production of various proinflammatory mediators, including cytokines, chemokines, nitric oxide, and prostaglandin E2 from immune or non-immune cells [12, 29–31]. As is ubiquitously expressed by a variety of cell types of multiple tissues, MIF participates in the pathogenesis of many inflammatory and autoimmune diseases by overriding the immunosuppressive effects of glucocorticoids [29, 32]. In the neuropathological CNS, MIF is inducibly upregulated within neurons, astrocytes, microglia, and epithelial cells of the choroid plexus [33, 34]. It interacts with CD74 and/or CXCR2/4 receptors of immune-related cells to activate intracellular ERK1/2 and AKT/PI3K signal pathway, thereby to facilitate inflammation, reactive gliosis, demyelination, and neuronal apoptosis [30, 35, 36]. Suppression of MIF following SCI significantly attenuates neuronal death and promotes functional recovery [15, 31]. So, MIF acts as one of the core DAMPs in exacerbating neuropathology. However, the mechanism about SCI-induced expression of MIF within specific cell type remains elusive.

Hemorrhage following breakage of BSCB always induces the activation of thrombin, a serine protease responsible for hemostasis by converting soluble plasma fibrinogen into an insoluble fibrin clot and by promoting platelet aggregation [37]. In addition to its effects on coagulation, thrombin has been shown to perform multiple pathological functions, including neuroinflammation [38, 39], astrogliosis [40, 41], demyelination [42], and other neurotoxicity [43, 44]. Thrombin-mediated cell events are mainly achieved by activation of one or more of the protease-activated receptor (PAR1, 3, 4) members through cleavage of the extracellular N-terminus to promote intracellular signaling [45, 46]. Although astrocytes in the CNS express all the four subtypes of PARs [47], PAR1 represents an efficient target to alleviate the proinflammatory and neurotoxic impact mediated by the protease [48, 49]. Given that thrombin/PAR1 axis contributes to proinflammatory glial signatures and functions immediately after CNS damage, it is conceivable that thrombin possibly promotes the production of DAMPs from the astrocytes. In the present study, we analyzed the correlations between activation of thrombin and the protein level of MIF in the astrocytes following spinal cord contusion. Transcriptome sequencing and in vitro model further identified that thrombin-mediated MIF expression in the astrocytes was regulated by MAPKs/NFκB signaling. Inhibition of thrombin following SCI significantly reduced MIF production and promoted functional recovery of rats. Our results have revealed the lead activator of SCI-induced DAMPs and also provided the crucial target for interfering neuroinflammation.

## Methods

### Animals

Adult male Sprague–Dawley (SD) rats, weighing 180–220 g, were provided by the Center of Experimental Animals, Nantong University. All animal experiments were approved by *the Animal Care and Use Committee of Nantong University* and the *Animal Care Ethics Committee of Jiangsu Province*. All rats were housed in standard cages (five rats in each cage) in an environment maintained at 22 ± 2℃ on a 12–12 h light–dark cycle and had free access to water and food.

### Establishment of contusion SCI rat model and drug treatment

The number of animals subjected to surgery was calculated by six per experimental group in triplicate. The contusion SCI rat model was prepared as the previous description [50]. In a nutshell, all animals were anesthetized with 10% chloral hydrate (300 mg/kg) administered intraperitoneally. The fur around the surgical site was shaved and the skin was disinfected with iodophor. Then the spinous processes of T8–T10 vertebrae were surgically exposed, and a laminectomy was performed at the ninth thoracic vertebral level (T9) with the dura remaining intact. The dorsal spinal processes T7 and T11 were fixed with the clamps of the impactor device (IH-0400 Impactor, Precision Systems and Instrumentation). The impactor rod was positioned centrally at T9 (about 3 mm in length) over the spinal cord midline, and the contusion was applied by receiving a 150-kilodyne contusion injury. The impact rod was removed immediately, and the wound was irrigated. The procedure was visually checked by formation of hematoma and by paralysis of the hindlimbs after animal awakening from the anesthesia. For drug delivery, a total of 4.5 μl of 5 mM PAR1 inhibitor SCH79797 (R&D systems) was slowly injected intrathecally, prior to the incision suture. As the vehicle for SCH79797 contains dimethyl sulfoxide (DMSO), which is not biologically inert, the 10 mM SCH79797 stock solution was prepared by addition of 1 mg of SCH79797 into 0.225 ml of 2.2 mg/ml DMSO dissolved in 0.1 M PBS. A double dilution (work solution: 5 mM SCH79797 in 0.1% DMSO) and a vortex were needed before use within 2 min. The rats were subcutaneously administered with a prophylactic dose of enrofloxacin (Sigma-Aldrich; 1 mg/kg) once daily for seven days to prevent urinary tract infection following surgery. Manual expression of bladders was performed twice a day until the animals recovered the spontaneous voiding.

### Cell culture and treatment

Astrocytes were prepared from the spinal cord of newborn SD rats, 1–2 days after birth, and the astrocytes were isolated and cultured according to the previously described methods [31]. Briefly, the spinal cords removed from the spinal canal were placed into 0.01 M PBS containing 1% penicillin–streptomycin. The spinal cord capsule was stripped clean under the microscope, followed by mincing with scissors and digestion with 0.25% trypsin for 15 min at 37 ℃. The digestion was terminated by addition of Dulbecco’s Modified Eagle’s Medium (DMEM)—high glucose medium containing 10% fetal bovine serum (FBS), 1% penicillin–streptomycin, and 1% L-glutamine. The suspension was then centrifuged at 1200 rpm for 5 min and the cells were resuspended and seeded onto poly-L-lysine pre-coated culture flask in the presence of 5% CO_2_. The medium was changed every 3 days until the whole flask is covered with cells. After 7–9 days, the culture flask was shaken at 250 rpm overnight to remove non-astrocytes. Astrocytic phenotype was evaluated by cell exhibiting a characteristic morphology and positive staining for the astrocytic marker glial fibrillary acid protein (GFAP). Astrocytes with purity more than 95% are acceptable for subsequent experiments.

To examine the effects of thrombin on MIF production from astrocytes, the serum was removed by washing 3 times during 15 min in serum-free DMEM. The cells were then subjected to stimulation with 0–200 nM rat thrombin (Sigma, T5772) in the presence or absence of selective inhibitor hirudin (Abcam), SCH79797 (R&D systems), tcY-NH_2_ (TOCRIS), PDTC (Beyotime), SB203580 (TOCRIS), SP600125 (TOCRIS), or PD98059 (TOCRIS) for 24 h prior to assay.

For knockdown of PAR3 expression in the astrocytes, the cells were cultured on the six-well plates for 24 h, followed by transfection of PAR3 siRNA1 (sense strand 5'-CCA ACA UCA UAC UCA UAA U dTdT-3', antisense strand 5'-U ACA CGG AGG GUA UGA AGA dTdT-3'), or scramble siRNA (sense strand 5'-GGC UCU AGA AAA GCC UAU GC dTdT-3', antisense strand 5'-GC AUA GGC UUU UCU AGA GCC dTdT-3') with iMAX transfection reagent (Invitrogen) for 24 h. The astrocytes were subsequently stimulated by 100 nM thrombin for another 24 h before ELISA or Western blot assay.

### MTT assay

The primary astrocytes were planted in 96-well plates with 6 × 10^3^ cells/well and cultured in an incubator at 37 ℃ and 5% CO_2_ for 24 h. The cells were subsequently treated with different concentrations of SCH79797, tcY-NH_2_, or hirudin for 24 h, followed by addition of 100 μl MTT working solution (MTT:DMEM medium = 1:9) to each well in a 37 ℃ incubator for 4–6 h. Finally, 100 μl of 20% SDS solution was added for 20 h, and the absorbance was measured with a microplate reader at a 570 nm wavelength.

### Western blot

Protein was harvested from cells with a buffer containing 50 mM Tris (pH 7.4), 150 mM NaCl, 1% Triton X-100, 1% sodium deoxycholate, 0.1% SDS, and 1 mM PMSF, following treatment with thrombin for 24 h. Alternatively, protein was extracted from 1 cm spinal segments of injured site at 0 day, 1 day, 4 days, and 1 week following contusion (*n* = 6 in each time point). Samples were vortexed for 30 min and centrifuged at 12,000 rpm for 15 min. The supernatants were collected and stored at − 20 ℃ for use. Protein concentration of each specimen was measured by the BCA method to maintain the same loads according to the manufacturer’s instructions. Proteins were heated at 95 ℃ for 5 min, and 20 μg of each sample was electrophoretically separated on 10% SDS-PAGE gel, followed by transferring onto a polyvinylidene difluoride (PVDF) membrane. The membrane was blocked with 5% skim milk in Tris-buffered saline containing 0.1% Tween-20 for 1 h, and then an overnight incubation at 4 ℃ with primary antibodies: MIF (1:500, Abcam); p65NFκB (1:1000, CST); ERK (1:1000, CST); p-ERK (1:1000, CST); JNK (1:1000, CST); p-JNK (1:1000, CST); P38 (1:1000, CST); and p-P38 (1:1000, CST). After washing 3 times with TBST for 10 min each, the membrane was incubated with secondary antibody goat anti-mouse HRP or goat anti-rabbit HRP (1:1000, Beyotime) for 2 h at room temperature. The HRP activity was detected using an ECL kit. The image was scanned with a GS800 Densitometer Scanner (Bio-Rad), and the data were analyzed using PDQuest 7.2.0 software (Bio-Rad). The β-actin (1:5000) was used as an internal control.

### ELISA

The primary cultured astrocytes were harvested after treatment with various drugs for different time. As for the measurement of MIF in the cord tissue samples, the contusion model was established as mentioned above, and the PAR1 inhibitor SCH79797 or vehicle was injected intrathecally before the incision was sutured. Spinal cord samples (*n* = 6 in each time point) at 1 cm length around the lesion site were harvested on 0 day, 1 day, 4 days, and 1 week after the rats were anesthetized with 10% chloral hydrate (300 mg/kg) and transcardially perfused with saline. Each sample weighed about 0.03–0.04 g. The cells or tissue samples were sonicated using the lysis buffer supplemented with a protease inhibitor PMSF. Homogenate was centrifuged at 12,000 rpm for 15 min at 4 ℃, and the supernatant was collected for MIF (Elabscience) and thrombin (Elabscience) ELISA assay. The concentrations of MIF and thrombin are expressed as ng/ml for the supernatant, while ng/mg for the cord tissues. Plates were read with a multifunctional enzyme marker (Biotek Synergy2) at a 450 nm wavelength.

### Tissue immunofluorescence

The animals were intraperitoneally anesthetized at desired time points as mentioned above following SCI and drug administration. Then, they were transcardially perfused with 0.01 M PBS (pH 7.4) and 4% paraformaldehyde. The vertebra segments were harvested from six experimental subjects of each time point, post-fixed with 4% paraformaldehyde in PBS at 4 °C overnight. After they were equilibrated in 10%, 20%, and 30% gradient sucrose, about twenty cord sections at 10 μm each were prepared using a cryostat from 0.25 cm length to the lesion epicenter. The sections were blocked with 0.01 M PBS containing 3% BSA, 0.1% Triton X-100, and 10% normal goat serum for 1 h at 37 ℃, and incubated overnight at 4 ℃ with primary antibodies: GFAP (1:400, Sigma); MIF (1:200, Abcam); and PAR1 (1:200, Novusbio)S100β (1:400, Abcam). Thereafter, the sections were rinsed with PBS and incubated with the Cy3-labeled goat anti-rabbit IgG (1:400, Proteintech) or the Alexa Fluor 488-labeled donkey anti-mouse IgG (1:400, Abcam). Sections were observed under a fluorescence microscope (ZAISS, axio image M2).

### Sequencing of mRNA

Total RNA of astrocytes following treatment with 100 nM thrombin for 6 h, 12 h, and 24 h, respectively, was extracted using the mirVana miRNA Isolation Kit (Ambion, Austin, TX) according to the manufacturer’s instructions. They were then selected by RNA Purification Beads (Illumina, San Diego, CA), and undergone library construction and RNA-seq analysis. The library was constructed by using the Illumina TruSeq RNA sample Prep Kit v2 and sequenced by the Illumina HiSeq-2000 for 50 cycles. High-quality reads that passed the Illumina quality filters were kept for the sequence analysis.

### Bioinformatics analysis

Differentially expressed mRNA was designated in a criterion of greater or less than twofold changes in comparison with the control. Function of genes was annotated by Blastx against the NCBI database or the AGRIS database with E value threshold of 10^–5^. Gene ontology (GO) classification was obtained by WEGO via GO id annotated by Perl and R program. Kyoto Encyclopedia of Genes and Genomes (KEGG) pathways were assigned to the sequences using KEGG Automatic Annotation Server (KAAS) online. For all heatmaps, genes were clustered by Jensen–Shannon divergence.

A reconstructed gene network was created using the Ingenuity Pathway Analysis (IPA) Software on the basis of differentially expressed genes to investigate their regulatory pathways and cellular functions.

### Polymerase chain reaction (PCR)

Total RNA was prepared with Trizol (Sigma) from cells treated with PAR3-siRNA (Ribobio) using a LipofectamineTM RNAiMAX transfection reagent (Invitrogen) for 24 h. Fluorescence-tagged control Cy3 was used as a marker for evaluation of transfection efficiency. Alternatively, total RNA was extracted from 1 cm spinal segments of injured site at 0 day, 1 day, 4 days, and 1 week following contusion (*n* = 6 in each time point). The first-strand cDNA was synthesized using HiScript II Q RT SuperMix for qPCR (+ gDNA wiper) (Vazyme) in a 20 µl reaction system, and diluted at 1:3 before used in assays. The sequence-specific primers were designed and synthesized by Ribobio. Primer pairs for *par1, par3, par4, and gapdh* are as follows: forward primer 5'-ACT TCA CCT GCG TGG TCA TCT G-3', reverse primer 5'-ATG GCG GAG AAG GCG GAG AA-3'; forward primer 5'-ATT GGT GTA CCA GCG AAC AT-3', reverse primer 5'-CGT TCC CAT TGA GAT GGT AG-3'; forward primer 5'-GTC AAC GCC TCA CCA CCA TAC T-3', reverse primer 5'-GGA GCC AGC CAA TAG GAA GGT C-3'; and for, forward primer 5'-GGG TCC CAG CTT AGG TTC AT-3', reverse primer 5'-GAG GTC AAT GAA GGG GTC GT-3', respectively. Endpoint PCR was performed with the specific primers and KOD-Plus-Neo (Totybo) master mix using a cycling program: 94 °C, 120 s; 98 °C, 10 s; 58 °C, 30 s; 68 °C, 60 s for 30 cycles on a thermocycler. A total of 10 μl of the PCR products was separated by electrophoresis on 1% agarose gel and the images were captured using Gel imaging system. For quantitative real-time PCR (Q-PCR), reactions were performed in a final volume of 10 μl (1 μl cDNA template and 9 μl reaction buffer containing 5 μl of 2 × ChamQ Universal SYBR qPCR Master Mix, 3 μl of RNase-free ddH_2_O, and 0.5 μl of anti-sense and sense primers each). Reactions were processed using one initial denaturation cycle at 94 °C for 5 min, followed by 40 cycles of 94 °C for 30 s, 60 °C for 30 s, and 72 °C for 30 s. Fluorescence was recorded during each annealing step. At the end of each PCR run, data were automatically analyzed by the system and amplification plots obtained. The expression levels were normalized to an endogenous *gapdh*. The relative expression was then processed using the 2^−ΔΔCT^ method. In addition, a negative control without the first-strand cDNA was also performed.

#### Hematoxylin–eosin (HE) staining

The spinal cord contusion model of the rat was prepared as mentioned above, and intrathecally injected with 4.5 μl of 5 mM SCH79797 or vehicle immediately. At 21 days after SCI the rats were anesthetized and transcardially perfused with 0.01 M PBS (pH 7.4) and 4% paraformaldehyde. The vertebra segments at 1 cm length around the lesion site were harvested from six experimental animals of each group, post-fixed with 4% paraformaldehyde, and equilibrated in gradient sucrose. The cord longitudinal cryosections at 12 μm were prepared across dorsal–ventral midline of the lesion epicenter. The sections were incubated with hematoxylin and eosin, followed by a process according to the standard procedure. Thereafter, the sections were observed under a fluorescence microscope (ZAISS, axio image M2). A total of three sections each animal were chosen for lesion quantification, based on the proportion of gray and white matter. The size of lesion area of the spinal cord before or after SCH79797 treatment was analyzed by NIH ImageJ software. For quantification of lesion area, a lesion border with 2000 μm rostral and caudal to the epicenter of the HE-colored sections was selected and suffered to image cropping by Adobe Photoshop software. Digital RGB images of the spinal cord were converted to 8-bit grayscale by ImageJ. After RGB color separation, the color thresholds were set with Image > Adjust > Threshold, so that the HE staining was colored red. HE area statistics (the area, area fraction expressed as %) were set up (Analyze > Set Measurements) prior to analysis. The lesion area was denoted by 1- HE%.

### Behavioral tests

The hindlimb locomotor function recovery was evaluated using the Basso, Beattie, and Bresnahan (BBB) locomotor scale as previously described [51, 52]. Briefly, after intrathecal injection of 4.5 μl of 5 mM SCH79797 or vehicle at 0, 7, 14, and 21 days, three well-trained investigators blind to the study were invited to observe the behavior of rats for 5 min. The BBB score ranged from 0 (no hindlimb movement) to 21 (normal movements, coordinated gait with parallel paw placement) according to the rating scale. Scores from 0 to 7 indicated the return of isolated movements in the hip, knee, and ankle joints. Scores from 8 to 13 meant the return of paw placement and coordinated movements with the forelimbs. Scores from 14 to 21 indicated the return of toe clearance during stepping, predominant paw position, trunk stability, and tail position. The tests were independently scored by the investigators following assessments.

### Statistical analysis

Statistical analysis used GraphPad Prism 8 software (San Diego, CA, USA). All data are presented as means ± SEM. Comparisons between two groups following of a normal distribution were analyzed by two-tailed unpaired Student’s *t* test or the Mann–Whitney test when the distribution was not parametric. Differences between multiple groups were analyzed using one­way or two-way analysis of variance (ANOVA), followed by Tukey’s or Sidak's post hoc test. *P* value < 0.05 was considered statistically significant and was denoted in the figures as *P* < 0.05.

## Results

### SCI-induced activation of thrombin parallels with the elevation of MIF protein at lesion sites

**Fig. 1:**
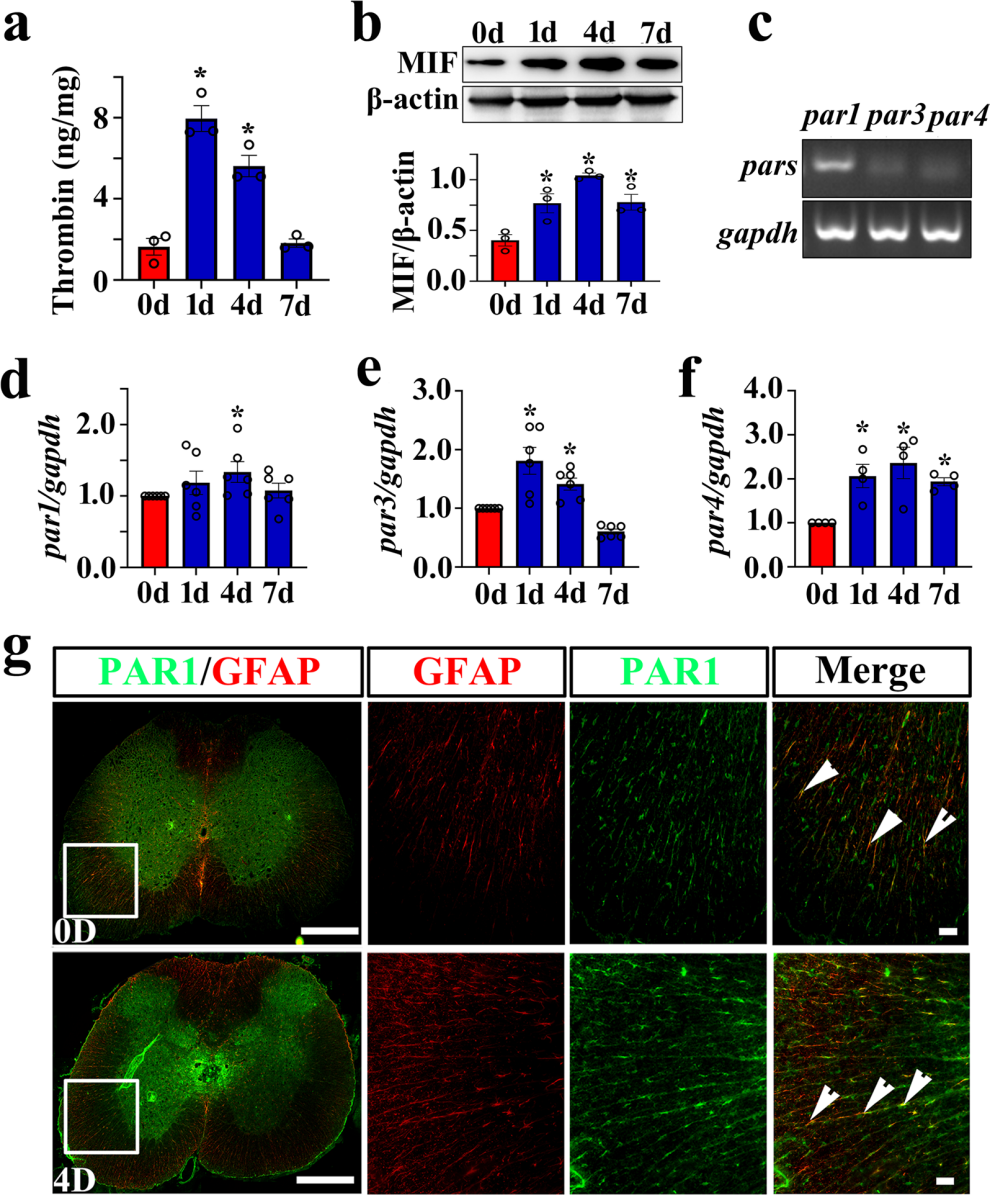
Determination of thrombin, PARs, and MIF expression at lesion sites following rat SCI. **a** ELISA measurement of thrombin protein levels at lesion sites following SCI at 0, 1, 4, and 7d. **b** Western blot analysis of MIF following SCI at 0, 1, 4, and 7d. Quantities were normalized to endogenous β-actin. **c** PCR assay for determining the expression abundance of *par1*, *par3,* and *par4* in the intact spinal cord. Quantities were normalized to endogenous *gapdh*. **d–f** RT-PCR assays of *par1* (**d**), *par3* (**e**), and *par4* (**f**) transcriptional changes following SCI at 0, 1, 4, and 7d, respectively. Quantities were normalized to endogenous *gapdh*. Experiments were performed at least in technical triplicates. The values shown in the figures were the average of each technical replicate. Error bars represent the SEM (*P < 0.05). **g** Colocalization of PAR1 with GFAP-positive astrocytes before and after SCI. The sections were prepared using a cryostat from 0.25 cm length to the lesion epicenter. Rectangle indicates region magnified. Arrowheads indicate the positive signals. Scale bars, 500 μm, and 50 μm in magnification

**Fig. 2:**
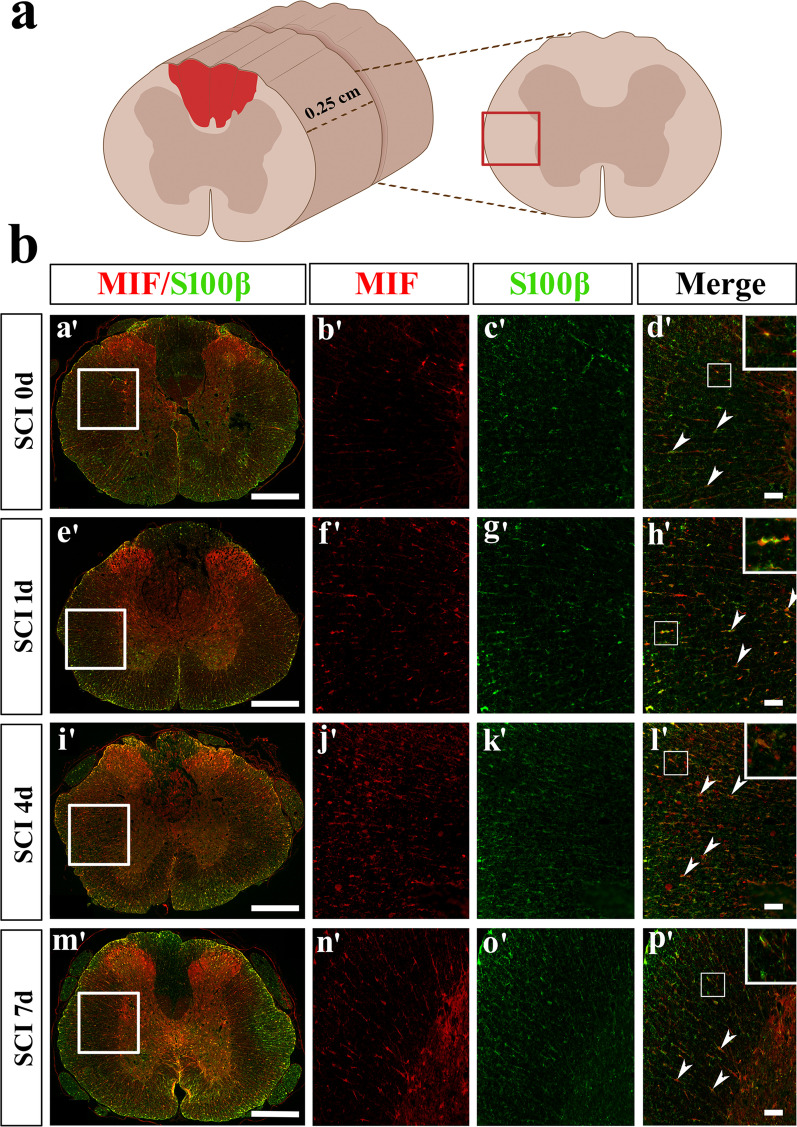
Colocalization analysis of MIF with S100β-positive astrocytes. **a** Illustration of section sites for immunostaining in the contused cord. **b** Colocalization analysis of MIF with S100β-positive astrocytes by immunostaining following SCI at 0, 1, 4, and 7d, respectively. Rectangle indicates region magnified. Arrowheads indicate the positive signals. Scale bars, 500 μm in **a’**, **e’**, **i’**, and **m’**; 50 μm in others

To explore the potential regulatory role of thrombin on the astrocytic production of MIF, the protein levels of the serine protease at lesion site were determined at 0d, 1d, 4d, and 7d following rat spinal cord contusion. ELISA results demonstrated that thrombin was significantly activated at 1d and 4d by the cord injury, but returned to the control level at 7d (Fig. 1a). Meanwhile, the protein levels of proinflammatory MIF in the injured tissues were inducibly increased at 1d, 4d, and 7d following SCI, as was determined by Western blots (Fig. 1b). It is known that thrombin mediates cell events through activation of PAR1, 3, and/or 4 receptor [47]. Thus, the semi-quantitative PCR was carried out to examine the transcriptional levels of PAR1, 3 and 4 receptors before and after SCI. The results demonstrated that the constitutive expression of PAR1 in the spinal cord was significantly higher than those of PAR3 or PAR4, with a slight upregulation at 4d following SCI (Fig. 1c, d). However, the expression of PAR3 and PAR4 was shown to be moderately induced by cord injury from 1d onwards, although at a relatively lower abundance (Fig. 1e, f). We next sought to observe the colocalization of PAR1 receptor with GFAP-positive astrocytes. Immunostaining displayed that PAR1 was detectable in the astrocytes before and after SCI (Fig. 1g). Further examination of MIF cellular distribution showed that this proinflammatory mediator was dynamically regulated by S100β-positive astrocytes at 0d, 1d, 4d, and 7d following cord contusion (Fig. 2a, b). The data indicate that SCI-induced elevation of thrombin possibly affects MIF production in astrocytes through activation of PAR receptors.

### Transcriptome sequencing manifests that thrombin dynamically regulates MIF expression of astrocytes

**Fig. 3:**
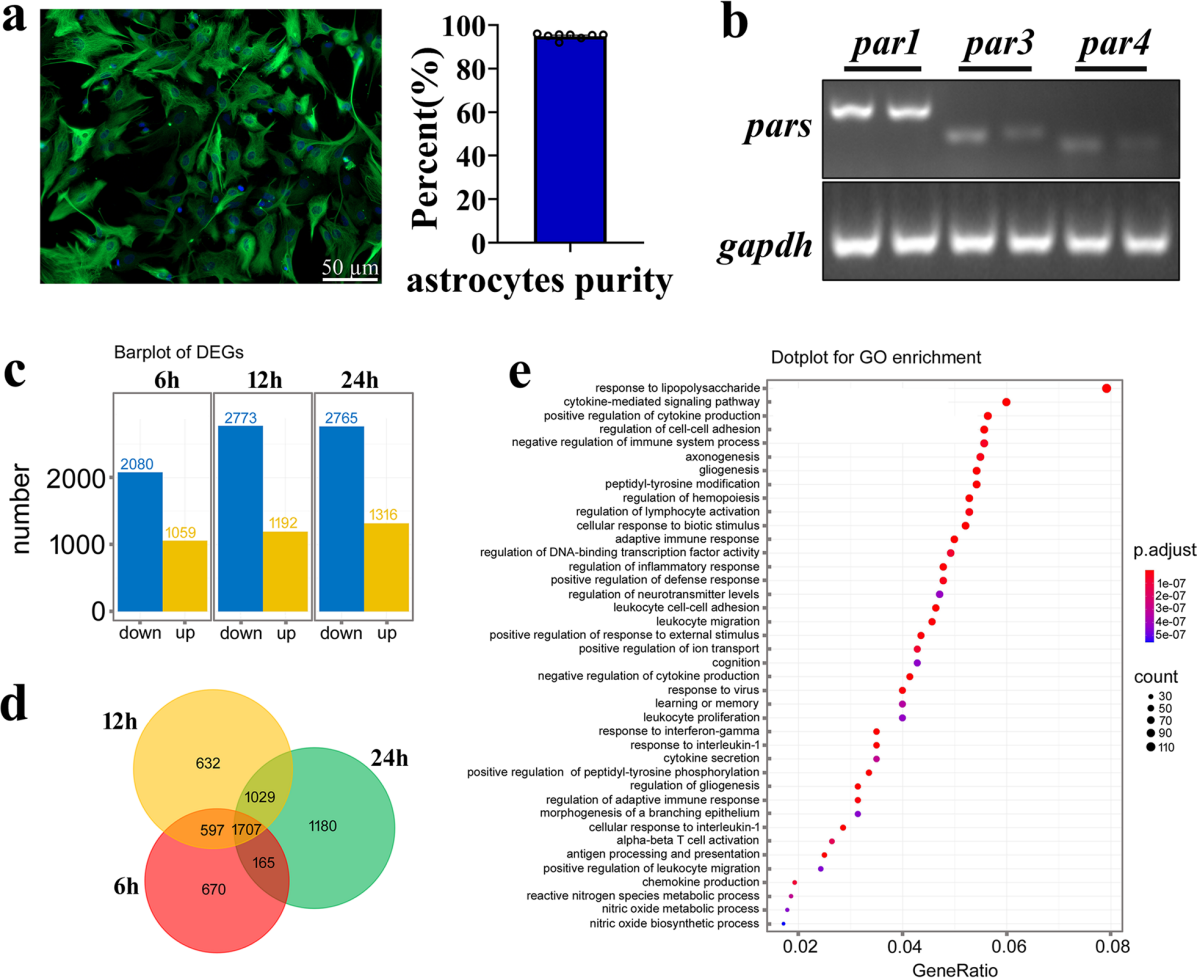
Functional annotation of DEGs following astrocyte stimulation with thrombin. **a** Primary cultured rat spinal cord astrocytes stained with GFAP and Hoechst 33,342 with purity over 95%. **b** PCR assay for determining the abundance of *par1*, *par3,* and *par4* at transcriptional levels in the primary astrocytes. Quantities were normalized to endogenous *gapdh*. c Bar graphs of DEGs following astrocyte stimulation with 100 nM thrombin for 6, 12, and 24 h, respectively. **d** Integration of DEGs at 6, 12, and 24 h. **e** GO analysis of the DEGs relating to biological processes. Scale bar, 50 μm in **a**

**Fig. 4:**
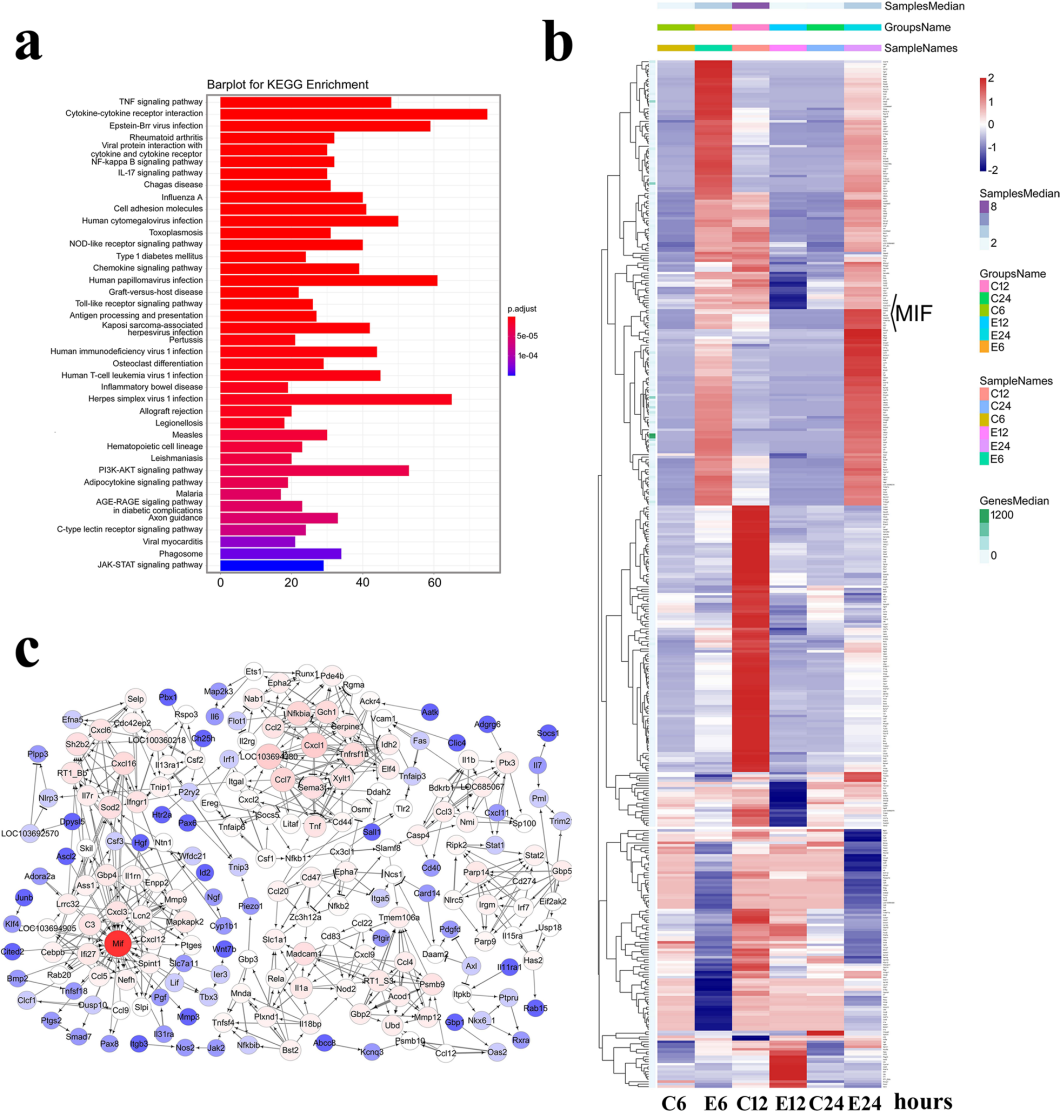
Expression profiling and gene network analysis of integrated DEGs following astrocyte stimulation with thrombin. **a** KEGG enrichment of the DEGs relating to signal pathways. **b** Heatmap and cluster dendrogram of integrated DEGs. **c** A reconstructed gene network was created using the IPA on the basis of integrated DEGs

To unveil thrombin-activated inflammatory pathways of astrocytes, the transcriptome sequencing was performed following stimulation of primary astrocytes with 100 nM thrombin for 6 h, 12 h, and 24 h, respectively. The purity of astrocytes isolated from spinal cord reached more than 95% (Fig. 3a). Semi-quantitative PCR determination recapitulated the high abundance of PAR1 expression in the primary cultured cells (Fig. 3b). Analysis of transcriptome profile revealed that a total of 3139, 3965 and 4081 differentially expressed genes (DEGs) were identified at the 3 time points, with defined criteria of *P* < 0.05 and a greater or less than twofold changes (Fig. 3c). Integration of these DEGs characterized 1707 functional genes that were dynamically regulated by thrombin (Fig. 3d). GO enrichment demonstrated that these integrated DEGs were involved in regulation of astrocytic biological processes, including response to LPS, cytokine-mediated signaling pathway, positive regulation of cytokine production, and cell–cell adhesion (Fig. 3e). KEGG analysis displayed that the functional relevant signal pathways, such as TNF-α signaling pathway, cytokine–cytokine receptor interaction and NFκB signaling pathway, were driven by these DEGs following thrombin stimuli (Fig. 4a). Heatmap and cluster dendrogram illustrated that these DEGs were dynamically regulated by thrombin at different time points (Fig. 4b). These data drawn from bioinformatics reflect that thrombin is able to activate astrocytic inflammation via modulation of multiple intracellular signals. To identify thrombin-induced MIF production in astrocytes, the ingenuity pathway analysis (IPA) was further performed on the DEGs integrated at 6 h, 12 h, and 24 h. A reconstructed gene network revealed that MIF was highlighted as a hub regulator with the highest weight value in response to thrombin stimuli (Fig. 4c). The results indicate that thrombin is a potential regulator of MIF expression in astrocytes.

### Thrombin efficiently facilitates the astrocytic production of MIF through PAR1/4 receptor

**Fig. 5:**
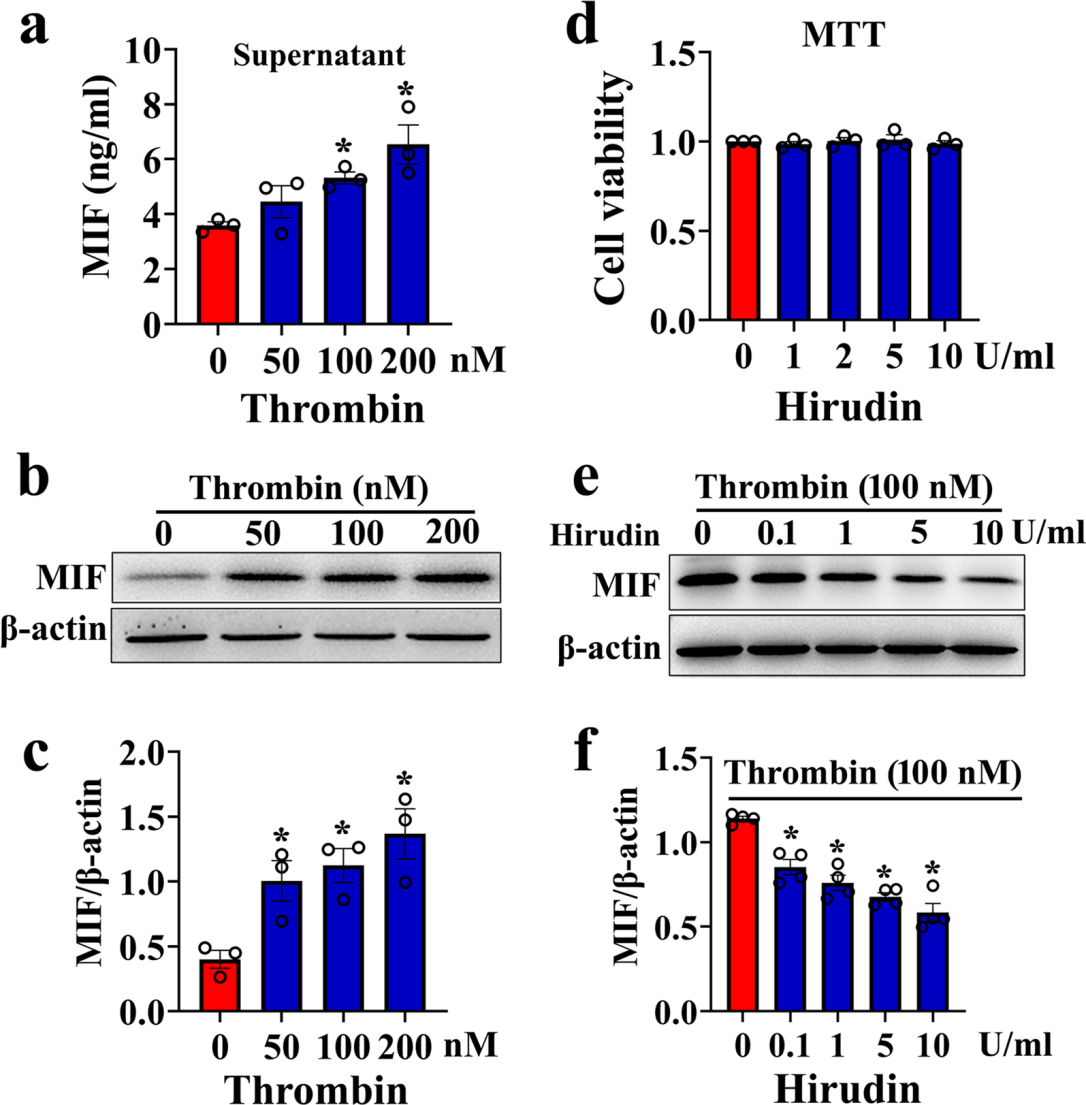
Effects of thrombin on the MIF production from astrocytes. **a** ELISA determination of MIF in the supernatants of primary astrocytes challenged by 0–200 nM thrombin for 24 h. **b** Western blot analysis of MIF protein levels in the astrocytes stimulated by 0–200 nM thrombin for 24 h. **c** Quantification data as shown in **b**. Quantities were normalized to endogenous β-actin. **d** MTT assay of hirudin effects on the cell viability of astrocytes. **e** Western blot analysis of MIF protein levels in the astrocytes treated by 0–10 U/ml hirudin in the presence of 100 nM thrombin. **f** Quantification data as shown in **e**. Quantities were normalized to endogenous β-actin. Experiments were performed in triplicates. Error bars represent the SEM (*P < 0.05)

**Fig. 6:**
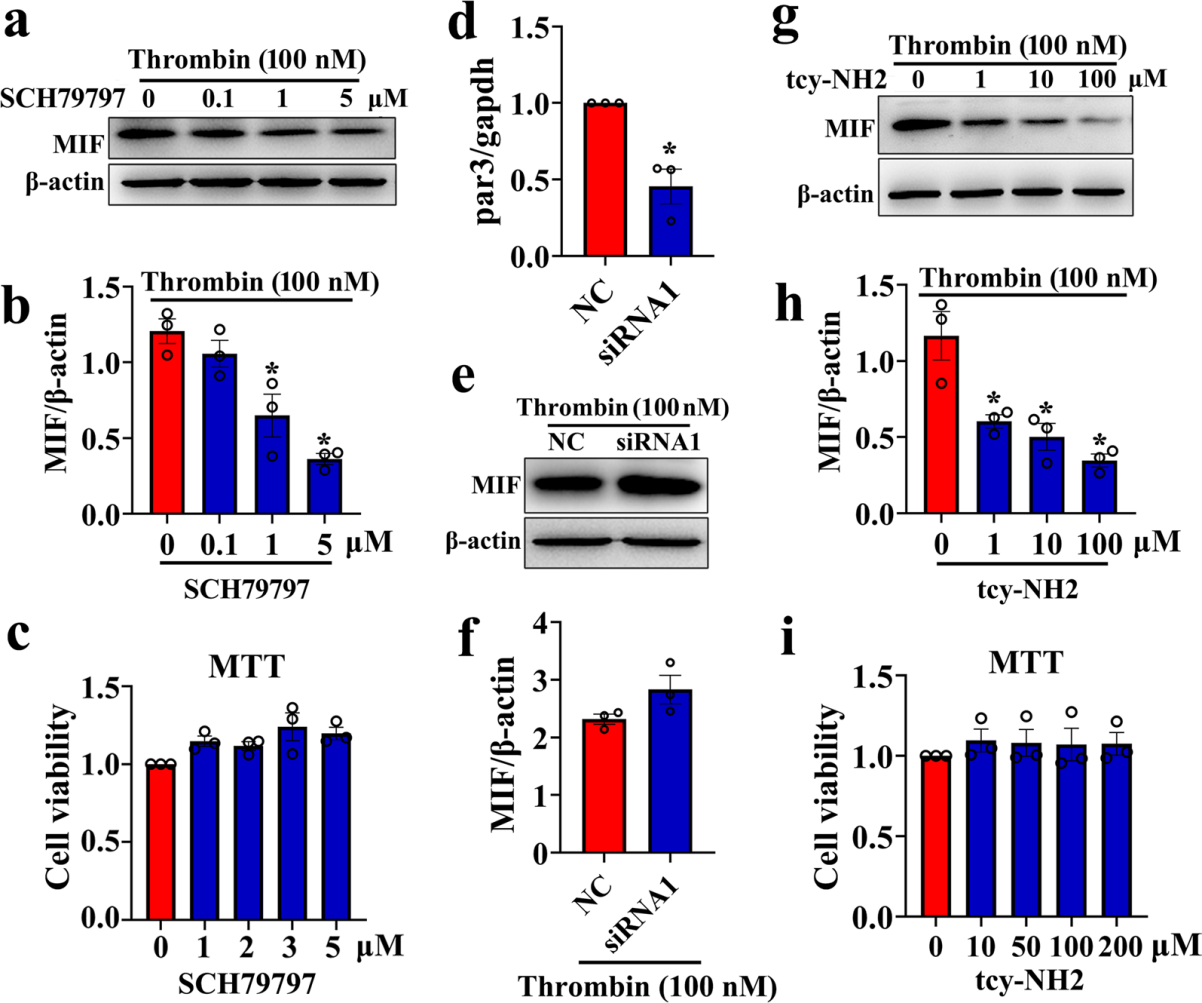
Effects of interfering PAR1, PAR3, or PAR4 expression on the thrombin-induced MIF production of astrocytes. **a** Western blot analysis of MIF protein levels in the astrocytes treated by 0–5 μM PAR1 inhibitor SCH79797 for 24 h in the presence of 100 nM thrombin. **b** Quantification data as shown in **a**. Quantities were normalized to endogenous β-actin. **c** MTT assay of SCH79797 effects on the cell viability of the astrocytes. **d** Interference efficiency of siRNA oligonucleotide for PAR3 was measured by RT-PCR, and siRNA1 was used for the knockdown experiments. **e** Western blot analysis of MIF in the astrocytes following PAR3 knockdown for 24 h, prior to stimulation with 100 nM thrombin for 24 h. Scrambles were used as control. **f** Quantification data as shown in **e**. Quantities were normalized to endogenous β-actin. **(g)** Western blot analysis of MIF protein levels in the astrocytes treated by 0–100 μM PAR4 inhibitor tcy-NH_2_ for 24 h in the presence of 100 nM thrombin. **h** Quantification data as shown in **g**. Quantities were normalized to endogenous β-actin. **i** MTT assay of tcy-NH_2_ effects on the cell viability of the astrocytes. Experiments were performed in triplicates. Error bars represent the SEM (*P < 0.05)

To validate the stimulatory roles of thrombin on the astrocytic production of MIF, the primary astrocytes cultured in serum-free medium were challenged with 0–200 nM thrombin for 24 h. ELISA and Western blot determination demonstrated that the protein levels of MIF in the cell supernatants and lysates were inducibly elevated by thrombin stimuli in dose dependence (Fig. 5a–c). Treatment of the cells with 0–10 U/ml selective inhibitor hirudin for 24 h in the presence of 100 nM thrombin significantly attenuated the production of MIF from astrocytes (Fig. 5d–f). As was expected, hirudin at 0–10 U/ml made no effects on the viability of the cells (Fig. 5d). To elucidate the exact PAR receptor(s) of thrombin action in regulation of MIF expression, the astrocytes were incubated with inhibitors of PAR1 or PAR4, or knocked down with PAR3 siRNA in the presence of 100 nM thrombin. The results demonstrated that addition of 0–5 μM PAR1 inhibitor SCH79797 or 0–100 μM PAR4 inhibitor tcy-NH_2_ to the culture medium for 24 h significantly alleviated the effects of thrombin-induced MIF production from astrocytes (Fig. 6a–c, g–i). MTT assay excluded the cellular toxicity of the two inhibitors (Fig. 6c, i). However, knockdown of PAR3 with efficient siRNA1 did not affect the protein level of MIF in astrocytes stimulated by 100 nM thrombin for 24 h (Fig. 6d–f). Taken together, thrombin is able to facilitate MIF production from astrocytes through activation of PAR1/4 receptor.

### Thrombin induces the astrocytic production of MIF through activation of intracellular MAPKs

**Fig. 7:**
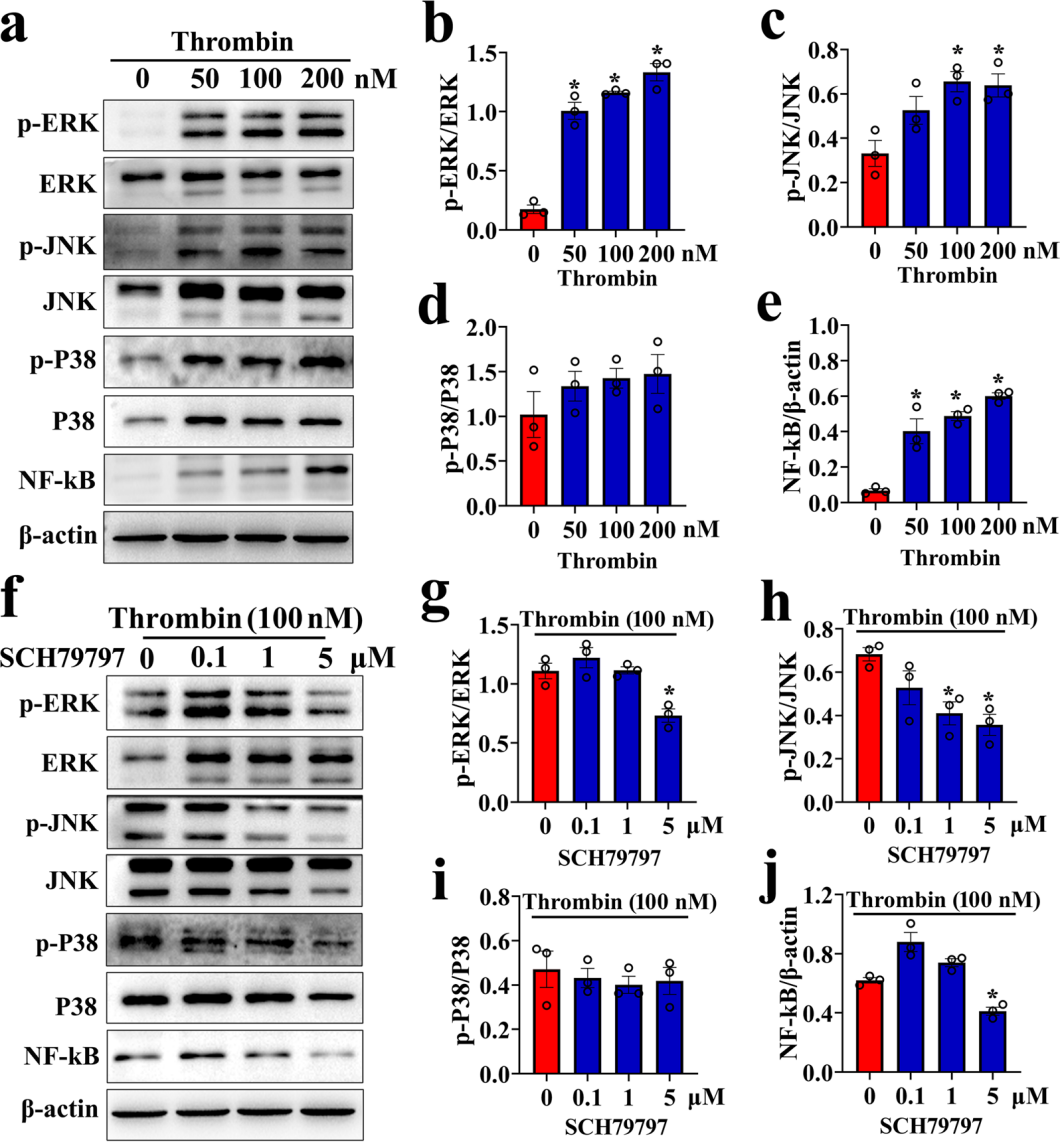
Determination of the phosphorylated activation of the MAPKs/NFκB signals in the astrocytes following stimulation with thrombin. **a** Western blot analysis of phosphorylation of ERK, P38, JNK kinase, and p65NFκB protein after astrocyte stimulation with 0–200 nM thrombin for 24 h. **b–e** Quantification data as shown in **a**. Quantities were normalized to endogenous β-actin. **f** Western blot analysis of phosphorylation of ERK, P38, JNK kinase, and p65NFκB protein after astrocyte treatment with 0–5 μM SCH79797 for 24 h in the presence of 100 nM thrombin. **g–j** Quantification data as shown in **f**. Quantities were normalized to endogenous β-actin. Experiments were performed in triplicates. Error bars represent the SEM (*P < 0.05)

Thrombin activates inflammatory astrocytic responses via multiple signal pathways, such as STAT3 and PLCε/NFκB pathways [43, 53]. Also, it has been shown to stimulate the activity of MAPKs [54]. To elucidate the mechanism of thrombin-mediated MIF production in astrocytes, the MAPKs/NFκB pathway was taken into primary consideration, due to its critical role in inflammatory activation of CNS [55]. Exposure of primary astrocytes to 0–200 nM thrombin for 24 h resulted in the dose-dependent increase of phosphorylated ERK, JNK, and p65NFκB protein levels, but not of P38 (Fig. 7a–e). Addition of 0–5 μM PAR1 inhibitor SCH79797 to the culture medium in the presence of 100 nM thrombin for 24 h significantly inhibited the stimulatory effects of thrombin on the phosphorylation of ERK, JNK, and p65NFκB, with the most efficient dose at 5 μM. However, the activation of P38 remained unaffected (Fig. 7f–j). The results indicate that thrombin is able to activate intracellular MAPKs/NFκB pathway of astrocytes.

**Fig. 8:**
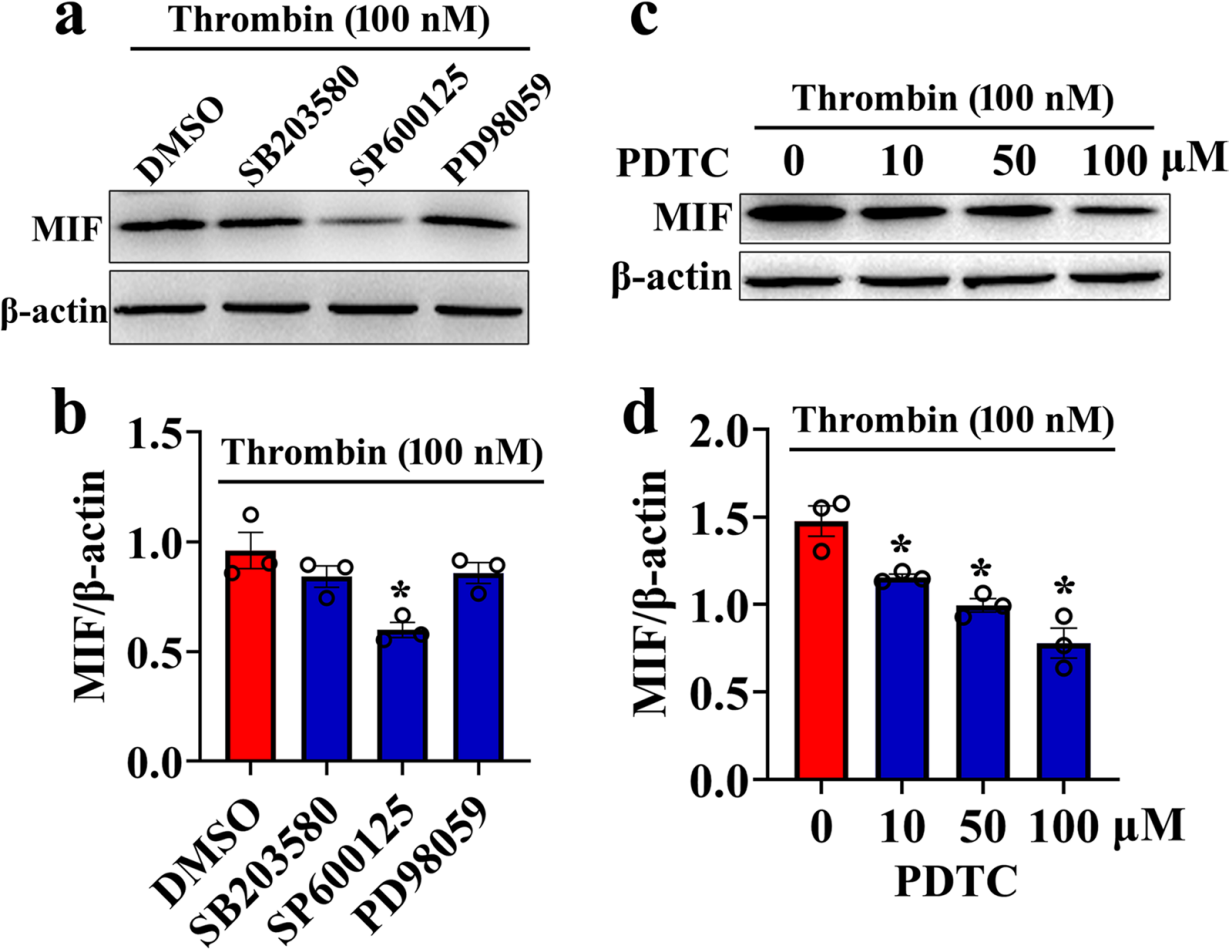
Effects of MAPKs and NFκB inhibition on the MIF production of astrocytes. **a** Western blot analysis of MIF following astrocyte treatment with 100 nM thrombin in the presence of 10 μM P38 (SB203580), 10 μM JNK (SP600125), or 10 μM ERK (PD98059) inhibitor for 24 h. **b** Quantification data as shown in **a**. Quantities were normalized to endogenous β-actin. **c** Western blot analysis of MIF following astrocyte treatment with 0–100 μM NFκB inhibitor PDTC in the presence of 100 nM thrombin for 24 h. **d** Quantification data as shown in **c**. Quantities were normalized to endogenous β-actin. Experiments were performed in triplicates. Error bars represent the SEM (*P < 0.05)

To further investigate the role of thrombin-mediated activation of MAPKs on the MIF production, the selective inhibitor of ERK (PD98059), JNK (SP600125), or P38 (SB203580) was thus used to incubate astrocytes in the presence of 100 nM thrombin. Results revealed that treatment of astrocytes with 10 μM JNK inhibitor SP600125 for 24 h was able to attenuate thrombin-induced production of MIF (Fig. 8a, b), while ERK inhibitor PD98059 and P38 inhibitor SB203580 did not. Accordingly, the protein level of thrombin-stimulated MIF in astrocytes was also reduced by 0–100 μM NFκB inhibitor PDTC in dose dependence (Fig. 8c, d). The data indicate that thrombin-activated JNK/NFκB pathway contributes to the astrocytic production of proinflammatory MIF.

### Administration of PAR1 selective inhibitor suppresses the injury-induced production of MIF and improves motor function following rat SCI

**Fig. 9:**
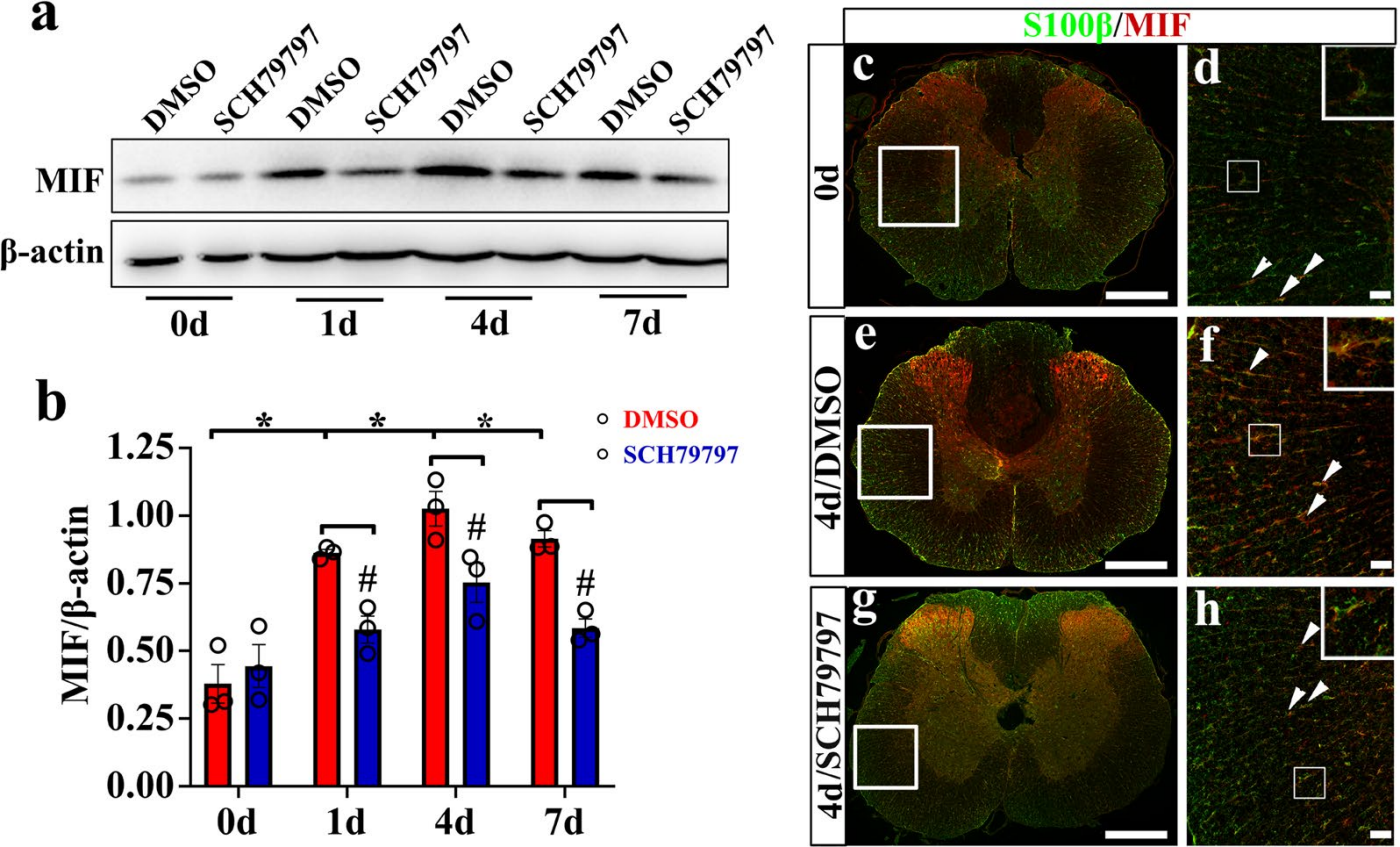
Effects of PAR1 inhibition on the production of MIF following rat SCI. **a** Western blot analysis of MIF from 1 cm cord segments following injection of 4.5 μl of 5 mM PAR1 inhibitor SCH79797 or vehicle at lesion sites of the contused cord at 0, 1, 4, and 7d, respectively. **b** Quantification data as shown in **a**. Quantities were normalized to endogenous β-actin. Experiments were performed in triplicates. Error bars represent the SEM, *P < 0.05, ^#^*P* < 0.05, two-way ANOVA with Tukey’s test. **c–h** Immunostaining for MIF expressed in S100β-positive astrocytes at 4d following SCH79797 treatment on the injured cord. Arrowheads indicate the positive signals. Scale bars, 500 μm in **c**, **e**, and **g**; 50 μm in **d**, **f**, and **h**

As SCI-induced production of MIF is involved in activation of innate immunity and worsens the neuropathology [15], the inhibitory effects of PAR1 on the production of MIF at lesion site, as well as on the functional recovery, were therefore evaluated. A total of 4.5 μl of 5 mM PAR1 inhibitor SCH79797 or vehicle was intrathecally injected at the lesion sites of the cords following contusion. Western blots demonstrated that SCI-induced elevation of MIF protein levels was significantly suppressed by the inhibitor at 1d, 4d, and 7d, in comparison with the control (Fig. 9a, b). Immunostaining was performed to detect the expression changes of MIF in the astrocytes before and after treatment of SCH79797, showing that MIF expression in S100β-positive astrocytes was remarkably decreased (Fig. 9c–h). These data indicate that inactivation of PAR1 is efficient in inhibition of thrombin-mediated MIF production from astrocytes following SCI.

**Fig. 10:**
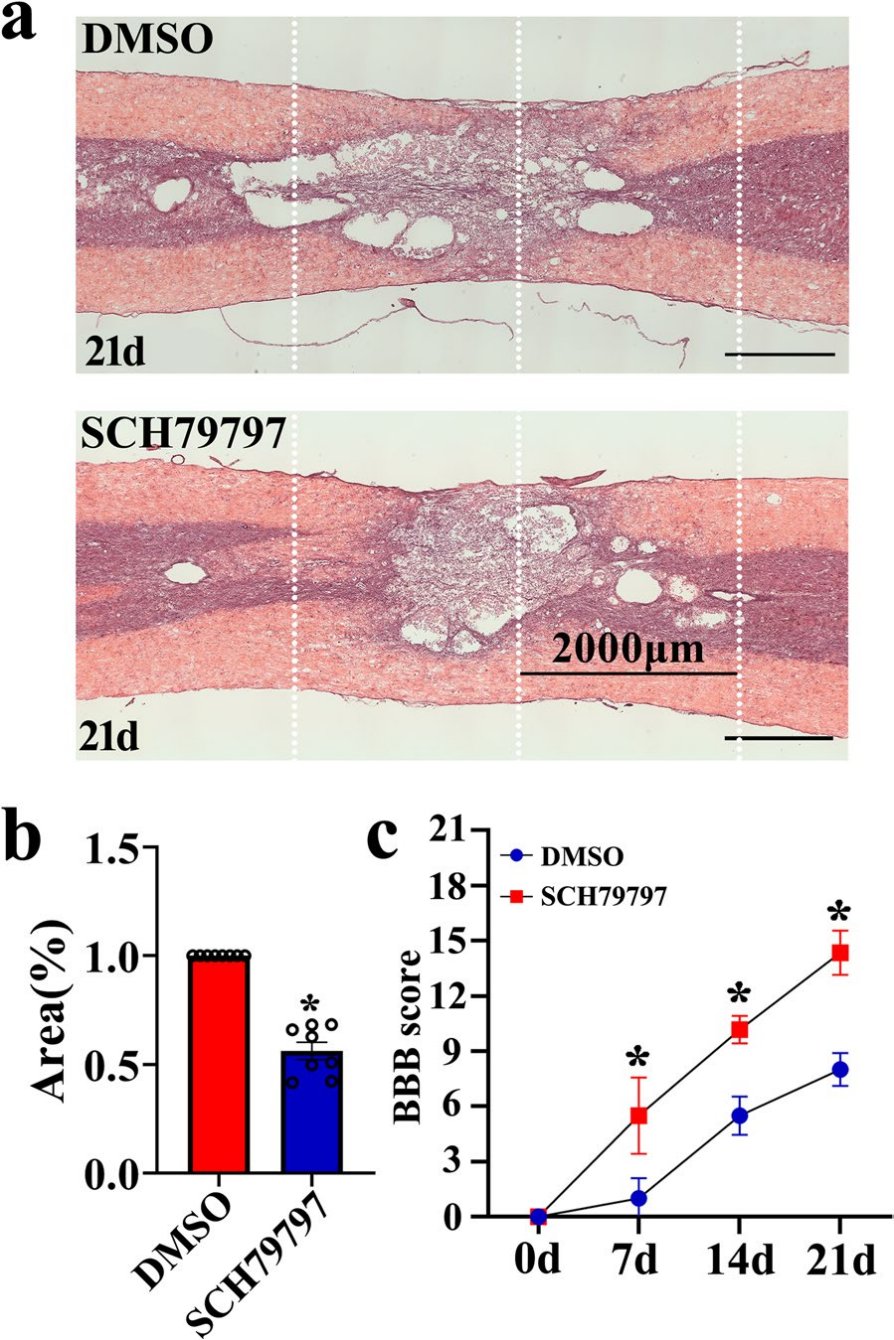
Effects of PAR1 inhibition on the recovery of motor function following rat SCI. **a** HE staining of the injured spinal cord at 21 d after injection of 4.5 μl of 5 mM PAR1 inhibitor SCH79797 or vehicle at lesion sites. **b** Quantification data as shown in **a** from eight animals each 3 sections. Lesion area was analyzed within 2000 μm either side of the lesion center based on the HE-negative proportion. **c** BBB score of hindlimbs analyzed two-way repeated measures ANOVA followed by Sidak's post hoc test at 0d, 7d, 14d, and 21d following intrathecal injection of 4.5 μl of 5 mM PAR1 inhibitor SCH79797 or vehicle at lesion sites. Error bars represent the SEM (*P < 0.05). Scale bars 1000 μm

Excessive inflammation always results in second tissue damage characterized by expansion of lesion size and severe loss of motor function. To shed light on the effects of SCH79797, which was shown efficient in suppressing proinflammatory MIF, on the functional recovery of rats after SCI, HE staining was performed to observe the damaged cord tissues. Results demonstrated that intrathecal injection of SCH79797 was able to reduce the lesion area of the cord at 21d following spinal cord contusion in comparison with the vehicle (Fig. 10a, b). BBB scores were subsequently assessed during 3 weeks to evaluate hindlimb locomotor function. Behavioral tests showed that inhibition of PAR1 immediately after SCI significantly improved the functional recovery of the rats (Fig. 10c). The data indicate that inhibition of thrombin-mediated production of MIF in astrocytes is able to ameliorate motor function following rat SCI.

## Discussion

DAMPs are critical initiators and amplifiers of innate immunity in insulted CNS [56]. They comprise diverse class of molecules, including bacterial lipids or peptides, endogenous proteins, nucleic acids, and metabolites [2]. Any insults or stress that threatens the homeostasis of CNS will result in their passive release by dead cells or active secretion by living cells [56], and their persistent production can elicit positive feedback of inflammation that eventually exacerbates damages to CNS tissue [57]. Most of the DAMPs are constitutively or endogenously expressed by cells to perform essential physiological functions; however, it is currently unknown what factors facilitate the secretion of the DAMPs from living cells following CNS insults. Indeed, many neurodegenerative diseases suffered from aberrant sterile inflammation are puzzled by the etiology [58, 59]. In the present study, we unveiled that thrombin was the culprit in mediating production of MIF from astrocytes. The findings will provide a novel clue for the therapy of MIF-induced pathogenesis of neurodegenerative diseases (AD) [60], autoimmune disorders (MS) [61], CNS tumor [62], as well as other inflammatory neuropathology [63]. As several cell types in the CNS, such endothelial cells and microglia, constitutively or inducibly express PAR1 receptor [64, 65], it is therefore assumed that the upregulation of MIF within these cells following SCI is, to some extent, attributed to the activation of thrombin. Whether the MIF-mediated peripheral inflammation, including rheumatoid arthritis and systemic lupus erythematosus, correlates with the aberrant activation of thrombin deserves further study. There are several lines of evidence showing that both thrombin and MIF severely affect the pathogenesis of the two diseases [66–69].

Although all the four PAR receptors are expressed by the astrocytes, thrombin cannot activate the trypsin receptor PAR2, but only be the agonist of PAR1, PAR3, and PAR4 in the astrocytes [70]. PARs are widely expressed in various organ systems with the most abundance of PAR1 in the CNS [47, 70]. Functional investigation demonstrates that PAR1 and PAR4 are the two human platelet thrombin receptors, whereas in astrocytes, PAR1, PAR3, and PAR4 are known to act as thrombin receptors in transduction of intracellular signals [47, 71]. Therefore, thrombin-mediated cell consequences are tightly associated with different cell types that express distinct PAR receptors. In the present study, it is interesting to note that activation of PAR1 and PAR4, rather than PAR3 receptor, is involved in promoting astrocytic expression of MIF. Previous studies reveal that activation of PAR1 will induce proliferation of astrocytes, while activation of PAR4 exerts toxic effects, suggesting the differential intracellular signals downstream of these two receptors are shared in regulation of MIF. PAR3 is thought to be a cofactor of the PAR4 receptor [72], and its activation is likely to be dispensable for the MIF production of astrocytes challenged by thrombin, as was shown by our studies.

Comparatively, PAR1 is the most abundant receptor among PARs expressed by the astrocytes [47]. Thrombin has been found to cleave PAR1 even at low concentration, but needs higher concentration to activate PAR3 and PAR4 [47]. These suggest that only thrombin/PAR1 axis is mainly responsible for the progressive neuropathology following SCI, although PAR4 can be activated by the serine protease [43]. Here, we found that administration of PAR1 inhibitor following SCI significantly attenuated production of MIF at lesion site and promoted functional recovery of hindlimbs, with the similar effects produced by MIF inhibitor 4-IPP [31]. The results indicate that thrombin-mediated MIF production by cleavage of PAR1 receptor plays equivalent neuropathological roles to those of thrombin, reemphasizing the importance of an immediate inactivation of thrombin in limiting excessive inflammation following SCI.

PAR-mediated downstream signal transduction pathways include, but not limited to, disassociated Gα and/or Gβ/γ subunits-activated PLC-β, PKC, Rho-GEFs, RhoA, and Rac-mediated MAPKs (ERK1/2, p38), PI3K/Akt, PLC-ε, and Src pathways [53, 70, 73]. Also, PARs can signal through G protein-independent β-arrestin to initiate RhoA, PI3K/Akt, and Src (ERK1/2) pathways. Several of the downstream effectors finally activate NFκB and regulate proinflammatory gene expression [53]. Our results displayed that thrombin facilitated astrocytic expression of MIF through activation of JNK/NFκB signal pathway, as validated by the application of their inhibitors. Thrombin-induced activation of JNK has been seen to mediate many cell events of astrocytes, such as promoting proliferation [74] and protecting the cells against apoptosis [75]. As such, thrombin activates intracellular signal pathways of astrocytes in a function-dependent manner.

In conclusion, SCI-induced activation of thrombin results in proteolytic cleavage of astrocyte PAR1 receptor, which activates NFκB protein via phosphorylation of JNK. The increased NFκB activity promotes the proinflammatory MIF production from astrocytes, which in turn mediates neuropathology as one of the crucial DAMPs.

## Data Availability

The datasets used and/or analyzed during the current study are available from the corresponding author on reasonable request.

## Abbreviations

ANOVA: Analysis of variance
CNS: Central nervous system
BBB: Blood–brain barrier
MIF: Macrophage migration inhibitory factor
DAMP: Damage-associated molecular pattern
PARs: Protease-activated receptors
PRRs: Pattern recognition receptors
EAE: Experimental autoimmune encephalomyelitis
NFκB: Nuclear factor kappa-B
PBS: Phosphate-buffered saline
MAPK: Mitogen-activated protein kinases
TLRs: Toll-like receptors
SCI: Spinal cord injury
GFAP: Glial fibrillary acid protein
KEGG: Kyoto Encyclopedia of Genes and Genomes
IPA: Ingenuity Pathway Analysis
Q-PCR: Quantitative real-time PCR
BBB assessment: Basso, Beattie, and Bresnahan assessment
ELISA: Enzyme-linked immunosorbent assay
